# Granular Cell Pituitary Tumor in a Patient with Multiple Endocrine Neoplasia-1

**DOI:** 10.7759/cureus.4541

**Published:** 2019-04-25

**Authors:** Arjun V Pendharkar, Chieh-Yu Lin, Donald E Born, Andrew R Hoffman, Robert L Dodd

**Affiliations:** 1 Neurosurgery, Stanford University School of Medicine, Stanford, USA; 2 Pathology & Immunology, Washington University in St. Louis, St. Louis, USA; 3 Pathology, Stanford University School of Medicine, Stanford, USA; 4 Internal Medicine - Diabetes & Endocrinology, Stanford University School of Medicine, Stanford, USA

**Keywords:** multiple endocrine neoplasia type 1, men-1, granular cell tumor, pituitary tumor

## Abstract

Multiple endocrine neoplasia type 1 (MEN-1) is an autosomal dominant disorder characterized by parathyroid, pancreatic islet, and pituitary tumors. Approximately 40% of MEN-1 patients harbor a pituitary adenoma. Separately, granular cell tumors (GCTs) of the sellar/parasellar region are an exceedingly rare clinical entity with less than 100 reported cases in the literature. These slow-growing, often asymptomatic lesions are difficult to diagnose and may mimic pituitary adenoma, Rathke cleft cyst, or other sellar/supra-sellar pathology. There is no known association with MEN-1 or any other familial syndrome. A 36-year-old neurologically normal woman with known MEN-1 underwent a screening magnetic resonance imaging (MRI) scan which revealed a 10 mm x 6 mm x 7 mm sellar/suprasellar lesion. She underwent endoscopic endonasal transsphenoidal resection. Subsequent neuropathological analysis was consistent with GCT of the pituitary gland. Here we describe the first report to our knowledge of a GCT of the pituitary gland occurring in a patient with MEN-1.

## Introduction

Multiple endocrine neoplasia type 1 (MEN-1) is an autosomal dominant disorder characterized by parathyroid, pancreatic islet, and pituitary tumors. Approximately 40% of all MEN-1 patients harbor a pituitary adenoma [1].

Separately, granular cell tumors (GCTs) of the sellar/suprasellar region are an exceedingly rare clinical entity with less than 100 reported cases in the literature [2-4]. These slow-growing, often incidental and asymptomatic lesions are difficult to diagnose and may mimic pituitary adenoma [5], Rathke cleft cyst [6], or other sellar/supra-sellar pathology [7]. There is no known association of GCTs with MEN-1 or any other familial syndrome.

Here we describe the first report to our knowledge of a GCT of the pituitary gland occurring in a patient with MEN-1.

## Case presentation

**Figure 1:**
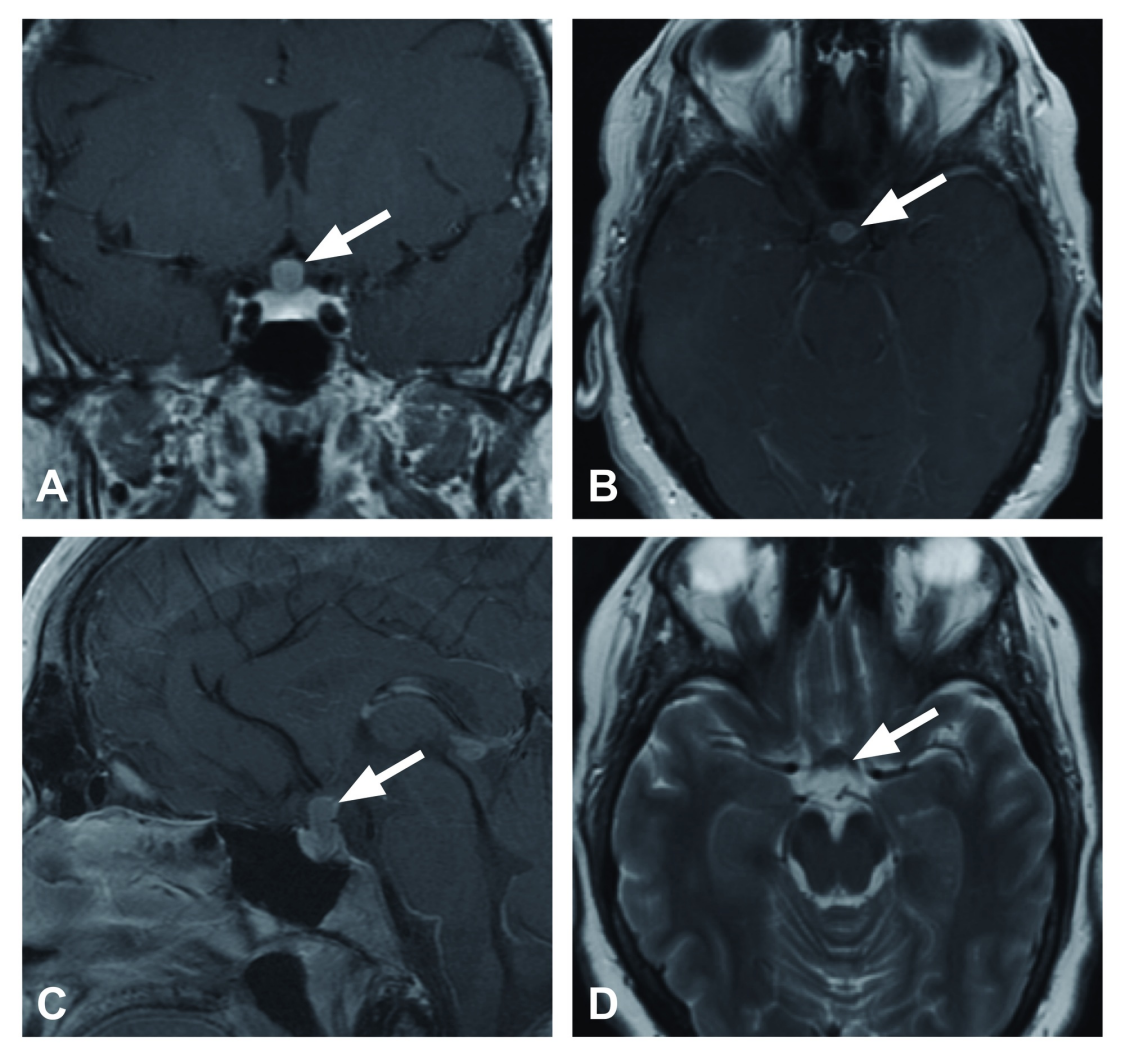
Preoperative magnetic resonance imaging. Preoperative magnetic resonance imaging demonstrates a hypoenhancing T1 and T2 intermediate lesion arising from the postero-superior aspect of the pituitary gland. The sellar-suprasellar lesion approximately 10 mm x 6 mm x 7 mm enhances with gadolinium administration. A) Coronal T1 post-gadolinium, B) axial T1 post-gadolinium, C) sagittal T1 post-gadolinium, D) axial T2.

The patient is a 36-year-old woman with MEN-1 status-post parathyroidectomy in whom a screening magnetic resonance imaging (MRI) scan demonstrated a 10 mm x 6 mm x 7 mm hypoenhancing T1 and T2- intermediate, contrast enhancing sellar/suprasellar lesion displacing and compressing the optic chiasm superiorly. The imaging was felt to be atypical for pituitary adenoma, and it remained unclear whether the lesion was involved with, or separate from the pituitary infundibulum (Figures 1A-1D). The patient was neurologically normal with intact visual fields and normal endocrine function. 

Given the possibility of future visual loss and the benefit of tissue diagnosis and given the unclear MRI findings, the patient elected for endoscopic endonasal transsphenoidal resection. Intraoperatively, a mass was clearly delineated posterior to the pituitary gland, adherent to the arachnoid tissue, and intraoperative frozen section confirmed neoplastic tissue. Gross total resection was achieved. The patient’s postoperative course was significant for development of panhypopituitarism and she was discharged on a standing regimen of cortisol, levothyroxine, and desmopressin.

**Figure 2:**
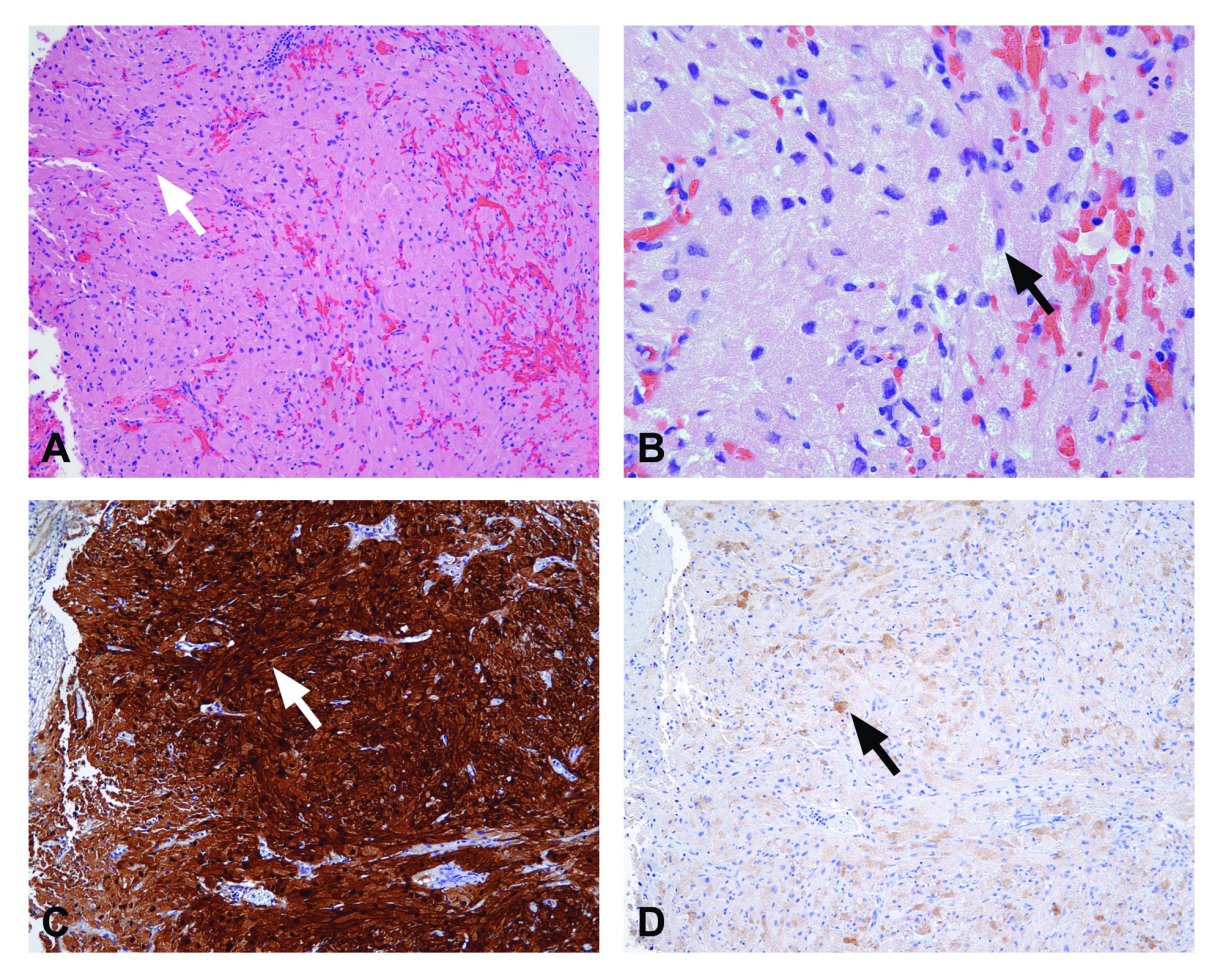
Neuropathological analysis. Morphologic and immunophenotypic characteristics are consistent with granular cell tumor of the pituitary gland. Proliferation of ovoid and spindle cells in loose nests and fascicles with indistinct cytoplasmic borders. Abundant eosinophilic granules are present in the cytoplasm while nuclei exhibit moderate atypia and are variable in size. A) Granular cell tumor 10x, B) granular cell tumor 40x, C) S100 10x, D) epithelial membrane antigen 10x.

Subsequent neuropathological analysis was consistent with GCT of the pituitary gland (Figures 2A-2D). Histological sections demonstrated proliferation of large ovoid and spindle cells in nests with abundant eosinophilic cytoplasmic granules. Immunohistochemistry revealed S100, chromogranin A, and thyroid transcription factor 1 (TTF-1) uniformly positive reactivity and variably positive epithelial membrane antigen (EMA) staining.

**Figure 3:**
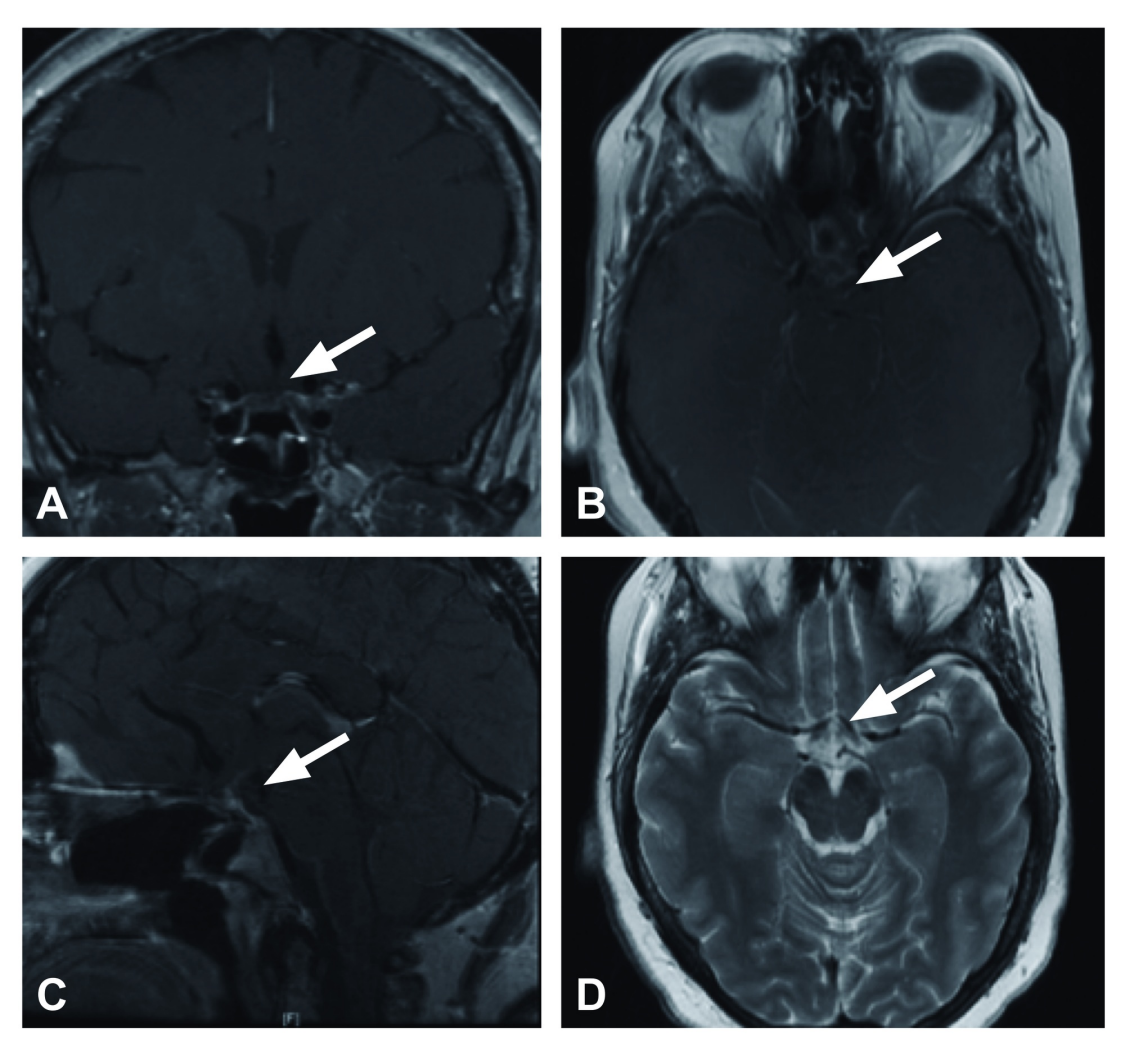
Postoperative magnetic resonance imaging at six months. Magnetic resonance imaging at six months with post-surgical changes and no residual or recurrent disease. A) Coronal T1 post-gadolinium, B) axial T1 post-gadolinium, C) sagittal T1 post-gadolinium, D) axial T2.

At one year follow-up, the patient was doing well clinically, neurologically normal with full visual fields maintained on a stable pituitary hormonal replacement. The MRI at that time demonstrated expected postoperative changes but no evidence of residual or recurrent disease (Figures 3A-3D).

## Discussion

Boyce and Beadles first reported an ‘accessory body taking origin from the infundibulum’ in 1893 and Sternberg first identified a granular cell tumor in 1921 [8-9]. These slow-growing, often asymptomatic lesions have previously been referred to as choristoma, pituicytoma, myoblastoma, and infundibuloma reflecting the confusion surrounding posterior pituitary and infundibular pathology. Currently GCTs are defined as WHO Grade 1 lesions originating from pituicytes and are a distinct clinical entity from pituicytoma [10]. Radiographically GCTs are most commonly hyperattenuated to cerebral parenchyma on computed tomography (CT) with a homogenous pattern of enhancement. The MRI reveals hypointense or isointense T1 signal and isointense T2 signal. GCTs tend to homogenously enhance with gadolinium contrast administration [11]. The histological features include nests of large cells with granular eosinophilic cytoplasm [12].

Although less than 100 cases have been reported in the literature, autopsy studies suggest that GCTs may exist in up to 9% of patients without any symptoms [13]. The most common presenting symptoms in clinical series are visual disturbance and headache and GCTs may mimic adenoma, Rathke’s cleft cyst or present with endocrine disturbances including panhypopituitarism or acromegaly [2, 6, 14-15]. Surgical resection is the definitive treatment and patients most commonly experience postoperative pituitary dysfunction due to infundibular involvement of the lesion [7, 16].

Up to 40% of patients with MEN-1 harbor a pituitary adenoma. Prolactinomas represent the overwhelming majority of cases although growth hormone, adrenocorticotropic hormone (ACTH), co-secreting, and nonfunctioning adenomas are well described. Pituitary adenomas in MEN-1 are larger at presentation and more histologically aggressive than their sporadic counterparts [1, 17]. There are no prior reports of pituitary lesions other than adenoma in MEN-1 and GCTs are not associated with any familial or syndromic diseases. Interestingly, Cusick et al. in 1982 reported a ‘granular-cell pituicytoma’ now defined as GCT in a multiple endocrine neoplasia type 2 (MEN-2) patient [18]. Schultz et al. in 2001 reported a true pituicytoma in a patient with unproven but high phenotypic suspicion for MEN-1 [19].

We report the first case of GCT occurring in a patient with MEN-1. In addition to the classic pituitary adenomas, pancreatic neuroendocrine and parathyroid tumors, MEN-1 is also loosely associated with adrenal cortical tumors, pheochromocytoma, lipoma, angiofibroma, collagenoma, and meningioma. Additionally, Hall et al. recently reported bilateral ovarian granulosa cell tumors in a patient with a rare c.654 + 1 G>A MEN1 mutation [20]. We conducted genetic testing on our patient, who was found to harbor a different MEN-1 mutation (K517 c.1549A>T) resulting in a nonsense codon. The question remains, however, if this was a sporadic occurrence or a rare manifestation of MEN-1. In the former scenario, our patient will require ongoing surveillance for development of a pituitary adenoma.

## Conclusions

We describe the first report to our knowledge of a GCT of the pituitary gland occurring in a patient with MEN-1.
